# Addressing participation of women in maternal health care in light of its key knowledge correlates: findings from the Indian Sundarbans

**DOI:** 10.1186/1753-6561-6-S5-O27

**Published:** 2012-09-28

**Authors:** Pratishtha Sengupta

**Affiliations:** 1 Future Health Systems - Institute of Health Management Research, Kolkata, India

## Introduction

According to the estimates by the World Health Organization (WHO), the reproductive ill health accounts for 33% of the total disease burden in women as compared to 12.3% for men. Despite this significant figure, in many South Asian countries the magnitude of reproductive morbidity has not been adequately defined. Maternal health event provides enough time to take requisite precautions and curative measures, if planned. In this conceptual paper we highlight the need to revisit the term literacy and its impact on maternal health from the angle of knowledge-cum-practice has been highlighted. Not only the ability to read and write but the actual learning has been examined that helps a woman and her households to perceive her illness and her ability to seek health services from the existing system.

## Methods

Primary data through case studies on specific maternal deaths as well as on high-risk maternal morbidity for two villages of the South 24 Paraganas district in Indian Sundarbans were collected under the scoping study of the Future Health System, a DFID supported cross-country program on innovative health planning for difficult regions.

We examined the knowledge and understanding of women and their families about their responsibilities in the pre-pregnancy, antenatal and postnatal care using this database. The questionnaire used also captured women’s linkage with groups, alliances, and social networks in and around the villages. In addition to this quantitative enumeration under sampled clusters, qualitative tools like focus group discussions with mothers, immediate relations, opinion leaders, and service providers were conducted exploring the issue of actionable knowledge to plan and prepare for healthy pregnancy.

## Results

Data have indicated that households with higher quintiles of literacy (60 percent considered as a cut off point) and that possess insignificant social linkages have in many instances failed to appreciate the importance of danger signs, plan and preparations during pregnancy. Households with lower literacy levels as a whole have better responded to the contingency call of maternal health once these are associated with support and information strength through networks and alliances or institutions like self help groups, *para unnayan samitis*, Village Health Sanitation Committees, and *Mahila* (women) committees.

Women’s community links were more strongly associated with postnatal care than that with antenatal care. To be precise, the three cases of maternal deaths in the last one year and a case of acute malnutrition in woman concerned have recorded none or negligible linkages with community networks and platforms.

Correspondingly, women having stronger participation in social life too have faced difficulties both during maternal and newborn care. However, in most cases they manage to avoid preventable hazards.

## Discussion

Leading data enumerating agencies like the Census of India, National Family Health Survey, Reproductive and Child Health-District Level Health Survey do collect data on literacy but there is still scope to address it from a more ‘practical’ approach. Knowing, understanding and utilizing the knowledge through existing institutions is something that translates literacy into real power and practice of knowledge.

This paper does not intend to come up with an exhaustive list of criteria upon which proposed revisiting on literacy definition would take place. Rather it calls for rethinking, reviewing, and formulating stronger prerequisites for addressing literacy from a more qualitative perspective that, in turn, becomes more relevant in understanding realization of maternal health entitlements that existing service delivery could offer.

## Competing interests

None declared.

## Funding statement

The study was funded by the Department For International Development (DFID), United Kingdom.

